# Impact of 8-week cold-and warm water swimming training combined with cinnamon consumption on serum METRNL, HDAC5, and insulin resistance levels in diabetic male rats

**DOI:** 10.1016/j.heliyon.2024.e29742

**Published:** 2024-04-16

**Authors:** Seyed Morteza Tayebi, Saleh Motaghinasab, Rasoul Eslami, Somayeh Ahmadabadi, Aref Basereh, Iman Jamhiri

**Affiliations:** a Department of Exercise Physiology, Faculty of Physical Education and Sports Sciences, Allameh Tabataba'i University, Tehran, Iran; b Department of Physical Education and Sports Sciences, Farhangian University, P.O. Box 14665-889, Tehran, Iran; c Faculty of Sports Science, Kharazmi University, Tehran, Iran; d Stem Cell Technology Research Center, Shiraz University of Medical Sciences, Shiraz, Iran

**Keywords:** Cinnamon, Diabetes, HDAC5, METRNL, Insulin resistance, Swimming

## Abstract

**Objective:**

Numerous studies have reported the beneficial effects of exercise and the use of herbal supplements in improving type 2 diabetes and insulin resistance. However, there are still many unanswered questions about the effects of cold and hot water, exercise, and herbal supplements on meteorine-like protein (METRNL), which is considered one of the key factors influencing insulin resistance improvement in this condition. Hence, the current study aimed to address these knowledge gaps and investigate the effects of 8 weeks of warm and cold-water swimming exercise with cinnamon consumption on serum levels of METRNL, histone deacetylase-5 (HDAC5), and insulin resistance in diabetic male rats.

**Methods:**

For this purpose, 70 diabetic male rats were randomly divided into seven groups (10 rats in each group) H ealthy control (HC) , Diabetic control , swimming training in cold water (temperature 5 °C) , swimming training at 5‌‌ °C + cinnamon consumption (200 mg/kg body weight) , swimming training in warm water (temperature 36-35 °C) , swimming training in warm water (temperature 36-35 °C) + consumption of cinnamon, and consumption of cinnamon only.

**Results:**

The present study revealed a significant increase in serum METRNL concentration in the cold-water swimming + cinnamon consumption group (p < 0.05). However, no significant changes were observed in insulin levels and HOMA-IR across the different groups (p > 0.05). Additionally, noteworthy findings included a significant reduction in HDAC5 levels in both the cold-water swimming group and the cold-water swimming + cinnamon consumption group, as well as a significant decrease in fasting blood sugar (FBS) levels in all groups compared to the HC group (p < 0.05).

**Conclusions:**

The results of the present study demonstrate that the combination of cold-water swimming exercises and cinnamon extract consumption led to notable increases in serum METRNL concentration. Additionally, significant reductions were observed in HDAC5 and FBS levels. These findings highlight the potential effectiveness and benefits of the combination of cold-water swimming exercises and cinnamon extract consumption as an approach to improve diabetes-related indices.

## Highlights

• Simultaneous use of physical activity, cold water immersion, and cinnamon supplementation in diabetic patients as a new method of therapeutic intervention.
• A significant increase in serum METRNL concentration in the cold-water swimming with cinnamon consume.
• Cold-water swimming with cinnamon consumption led to a significant decrease in HDAC5 and FBS levels.
• The combination of cold-water swimming exercises and cinnamon extract consumption as an approach to improve diabetes-related indices.

## 1 Introduction

Diabetes is a complex metabolic disorder characterized by elevated blood glucose levels, resulting from a disruption in insulin secretion, reduced insulin sensitivity, or a combination of both mechanisms [[1], [2], [3]]. Type 2 diabetes mellitus is commonly associated with obesity, a condition that can exacerbate insulin resistance, impairing the ability to utilize insulin effectively [4,5]. Adipose tissue releases adipokines, which are responsible for regulating various physiological processes including immune response, inflammation, energy balance, and insulin sensitivity [6]. One such adipokine is meteorin-like protein (METRNL), which is expressed in white adipose tissue and plays a role in influencing insulin sensitivity and the expression of GLUT4 [[7], [8], [9]]. By increasing intracellular calcium concentration, METRNL leads to increased phosphorylation of AMP-activated protein kinase (AMPK), and by activating phosphorylation of histone deacetylase-5 (HDAC5) by AMPK, it enhances the expression and transcription of GLUT4 [10]. Histone deacetylases (HDACs) are enzymes that play an important role in homeostasis. It has been shown that AMPK regulates the transcriptional activation of GLUT4 by controlling the nuclear export of HDAC5, which acts as a repressor of GLUT4 gene expression [11].

Physical activity and muscle contractions can increase glucose uptake in muscles through the transporter protein GLUT4, leading to improved insulin sensitivity [8]. The effect of exercise on adipose tissue and the secretion of adipokines [[12], [13], [14], [15]], particularly METRNL, has also been studied [8,9,16,17]. In an 8-week study involving both obese and normal mice, aerobic exercise was found to induce a substantial increase in METRNL levels within muscle and adipose tissue. Consequently, the exercise-induced elevation of METRNL in muscle effectively mitigated fat accumulation [16]. Recent research findings have indicated that following a single bout of exercise, there is a decrease in nuclear levels of HDAC5, leading to an increase in the expression of the GLUT4 gene [18]. Moreover, it has been observed that the nuclear translocation of HDAC5 is inversely correlated with GLUT4 expression. Thus, the upregulation of GLUT4 during exercise is linked to the downregulation of myocyte enhancer factor-2 (MEF2) and HDAC5 expression [19].

In addition, various stimuli such as cold water immersion and the use of certain supplements, such as cinnamon, have been found to modulate glucose transport. Studies have reported an increase in insulin sensitivity in mice subjected to cold exposure and an immediate reduction in plasma glucose concentration upon cold exposure in mice has also been observed [20]. Hanssen et al. (2015) reported a reduction in plasma glucose levels in individuals with type 2 diabetes following cold acclimation [21]. Research has revealed that during intense and moderate exercise, the rise in body temperature triggers an increase in appetite-suppressing hormones, subsequently leading to a decrease in appetite. Conversely, it appears that engaging in activities in cold water or under cold conditions can result in an elevated energy intake following the activity [22].

Therefore, given the direct association between diabetes and weight gain, as well as the influence of environmental temperature on appetite-related hormones during exercise, it is crucial to consider investigating physical activity under various temperature conditions.

Also, Cinnamon, a plant renowned for its insulin-stimulating and lipid-lowering effects [23], has been shown to have significant hypoglycemic and hypolipidemic effects at different doses per day [24]. Additionally, the findings from a study revealed that the combined intake of cinnamon extract and aerobic exercise over 8 weeks in hypertensive patients resulted in a notable decrease in levels of total cholesterol, fasting blood sugar (FBS), low-density lipoprotein (LDL), and total cholesterol (TC). Simultaneously, it was observed that insulin sensitivity significantly improved [25].

In another study, patients with type 2 diabetes were given a two-month supplementation of cinnamon, leading to a significant reduction in plasma glucose levels, triglycerides, and blood pressure [26]. Cinnamon has been shown to effectively modulate body temperature in cold temperature [24]. Li et al. (2020) discovered that the mechanism underlying cinnamon's ability to enhance cold tolerance is associated with the promotion of lipolysis and activation of brown adipose tissue (BAT) [24].

Given the observed positive effects of physical activity, cold water immersion, and cinnamon supplementation in diabetic patients, there is a need for additional research to explore the potential therapeutic applications of these interventions in diabetes management. Hence, this study aims to assess the impact of an eight-week cold-and-warm-water swimming exercise, along with concurrent cinnamon consumption, on the levels of METRNL, Homeostatic Model Assessment for Insulin Resistance (HOMA-IR), and HDAC5 in diabetic male rats.

## 2 Materials and methods

### 2.1 Animals

Seventy male rats, aged between 8 and 10 weeks, were procured from the animal breeding and reproduction center and acclimatized to the laboratory environment for one week in the animal physiology laboratory. Throughout the study, the rats were housed in transparent cages made of non-carbonate material, provided with standard rat food ad libitum, and maintained under controlled conditions of 12 h of darkness and 12 h of light, 55 % humidity, and a temperature ranging from 22 to 24 °C.

Following the initial acclimatization period, a total of 70 male rats were intraperitoneally injected with streptozotocin (Sigma, USA) at a dose of 55 mg/kg BW, Four days after the injection, blood glucose levels of the rats were measured through tail vein puncture. The diabetic rats were then categorized into seven groups for homogenization based on their fasting blood glucose levels. The study consisted of seven groups, namely [1]: Healthy control (HC) [2], Diabetic control (DC) [3], cold water swimming exercise (S5) [4], cold water swimming exercise with cinnamon extract supplementation (S5+Ci) [5], warm water swimming exercise (S35) [6], warm water swimming exercise with cinnamon supplementation (S35+Ci), and [7] cinnamon supplementation (Ci).

### 2.2 Experimental procedure

Initially, the rats underwent a one-week training period to assess their swimming ability in cold water. Each day, they were exposed to cold water for 2 min. Throughout this acclimation week, the water temperature was gradually lowered from the normal laboratory temperature of 25 °C–5 °C. The rats' behavior and activities were closely observed for 2 min, specifically noting their attempts to escape from the situation. To ensure familiarity with the exercise conditions, this procedure was repeated three times a week. Subsequently, swimming exercises were conducted at temperatures of 5 ± 2 and 35 ± 2 °C, following the protocol outlined by Lubkowska et al. (2019) [27].

During the initial week, the rats participated in 2-min exercise sessions for 5 days a week. Over time, the exercise duration was progressively increased by adding 30 s to each training session until reaching a total exercise duration of 4 min. After that until the end of the eighth week, the rats underwent 4-min swimming exercise sessions at a temperature of 5 °C. Additionally, a similar swimming exercise protocol was implemented at a temperature of 35 °C during the same time as the cold water swimming exercise. The swimming activities of the rats took place in a specialized swimming tank with dimensions measuring 100 cm in length, 50 cm in width, and 50 cm in depth [27,28].

### 2.3 Cinnamon supplementation

To prepare the cinnamon extract, 200 g of dry cinnamon powder was boiled in 1000 mL of distilled water for 10 min. After cooling, the solution was filtered through a No. 1 filter paper. The resulting solution had a concentration of 20 % cinnamon extract, meaning that each mL of the solution contained 20 mg of cinnamon extract.

In the cinnamon-supplemented groups, 1 mL of the prepared cinnamon extract was added to the drinking water for every 5 rats in each cage, which was approximately equivalent to a dosage of 1 kg of body weight.

Following this, the rats underwent an 8-week exercise regimen consisting of 5 sessions per week, following the prescribed exercise protocol. The 8-week training protocol was selected in the current study based on the previous study which showed that functional increases in physiological aspects resulting from exercise have been preserved after 8 weeks in diabetic and healthy individuals [29,30].

The rats in the cinnamon consumption group received a dosage of 200 mg/kg BW of cinnamon extract [31].

### 2.4 Data collection

After 48 h following the final training session, the rats were anesthetized using xylazine-ketamine, and their tissues were collected for analysis. Blood samples were obtained from the portal vein in fasting mode to avoid additional glucose from the intestines, and the plasma was separated by centrifugation at 3000 rpm for 10 min. These plasma samples were then sent to the laboratory for further measurements. The serum levels of METRNL were quantified using the ELISA-enzyme method with the Meteorin-like Protein kit manufactured by Zelbio, Germany. The sensitivity of this kit was 0.06 ng/mL.

The serum levels of HDAC5 were determined using the ELISA-enzyme method, employing the Histone Deacetylase 5 kit provided by Zelbio, Germany, which had a sensitivity of 0.06 ng/mL. Fasting glucose levels were assessed using the enzymatic method and the Hitachi902 autoanalyzer, utilizing the Pars Azmoon kit manufactured in Iran, which had a sensitivity of 5 mg/dL.

The measurement of serum insulin levels was performed using two different kits. Firstly, the Insulin kit (monobind) from a US-based company, employing the ELISA-enzyme method, with a sensitivity of 75 % μIU/mL. Secondly, the Pars Azmoon insulin kit (manufactured in Iran) was used with the same ELISA-enzyme method. The insulin resistance index (IR) was determined using the following formula:

$$\begin{array}{cc} \text{Glucoseinmassunits}\left(\text{mg}/\text{dL}\right) & \text{Glucoseinmolarunits}\left(\text{mmol}/L\right)\\ \text{HOMA}-\text{IR}=\frac{\text{Glucose}\times \text{Insulin}}{405} & \text{HOMA}-\text{IR}=\frac{\text{Glucose}\times \text{Insulin}}{22.5} \end{array}$$

### 2.5 Statistical analysis

The data analysis was conducted using the SPSS software, employing the inferential statistical method of one-way analysis of variance (ANOVA). It is conducted the Homogeneity of variances (Leven's test) and normality (Shapiro-Wilk's test) to ensure that the assumptions of one-way analysis for variance (ANOVA) were met and used the LSD's psot-hoc test for multiple comparisons. A significance level of p < 0.05 was considered statistically significant and deemed acceptable for this study.

## 3 Results

Leven's test showed that homogeneity of variances, and Shapiro-Wilk's test for data normality is met for all factors. So, one-way analysis for variance (ANOVA) is done for test of study hypotheses.

**Fig. 1:**
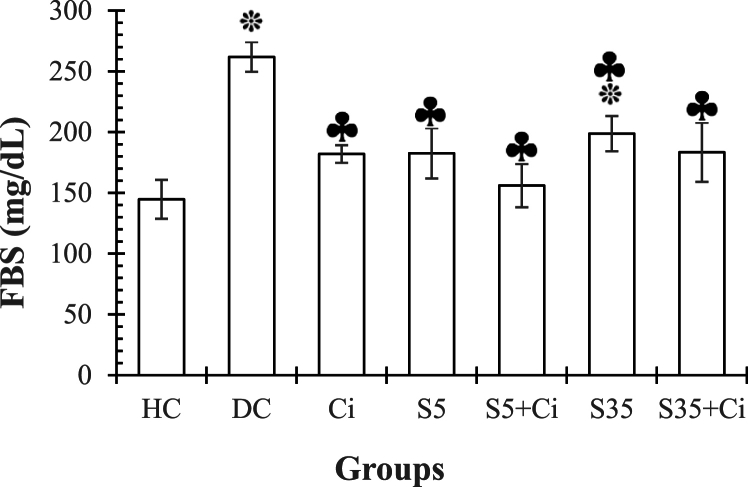
represents the Mean ± SE of FBS (Fast Blood Glucose) in different groups. *: significant differences compared to the HC group. **♣:** significant differences compared to the DC group. **HC:** Healthy Control. **DC:** Diabetic Control. **Ci:** Cinnamon feeding. **S5:** Swimming in 5⸰C water. **S35:** Swimming in 35⸰C water.

**FBS**. ANOVA revealed that the effect of groups was significant (F _(6,48)_ = 4.96, p < 0.001, η^2^ = 0.415). LSD's post-hoc test results showed that the glucose level in the DC and S35 groups was higher than in the HC group (p < 0.001, p < 0.0029, respectively), and in the Ci, S5, S5+Ci, S35, and S35+Ci groups was significantly lower than in the DC group (p = 0.002, p = 0.002, p < 0.001, p = 0.012, and p = 0.002, respectively). No significant differences were found between S5 and S35 groups (p > 0.05), and also between Ci, S5+Ci, and S35+Ci groups (p > 0.05) (Fig. 1).

**Fig. 2:**
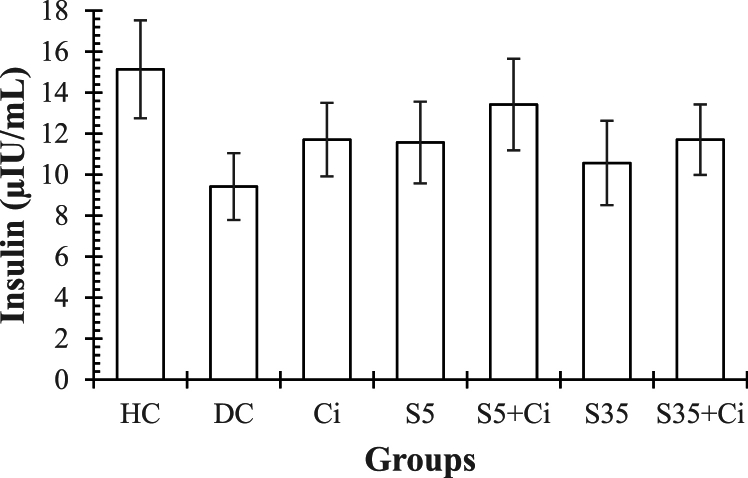
represents the Mean ± SE of Insulin in different groups. **HC:** Healthy Control. **DC:** Diabetic Control. **Ci:** Cinnamon feeding. **S5:** Swimming in 5⸰C water. **S35:** Swimming in 35⸰C water.

**Insulin.** ANOVA revealed that the effect of groups was not significant (F _(6,48)_ = 0.87, p = 0.521, η^2^ = 0.111)(Fig. 2).

**Fig. 3:**
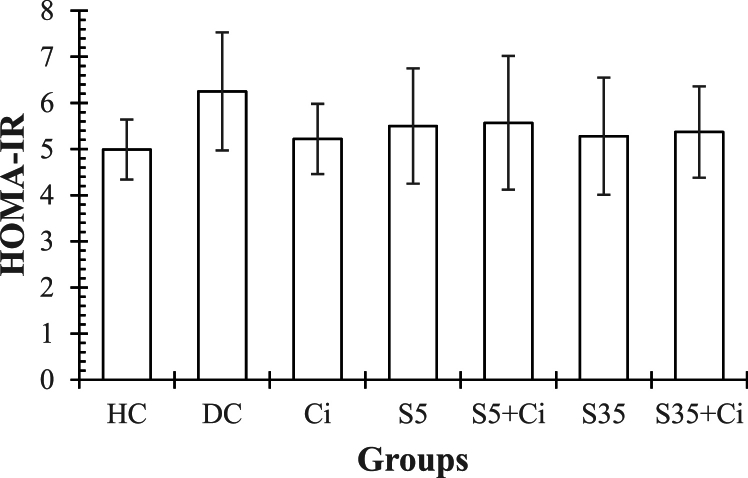
represents the Mean ± SE of HOMA-IR in different groups. **HC:** Healthy Control. **DC:** Diabetic Control. **Ci:** Cinnamon feeding. **S5:** Swimming in 5⸰C water. **S35:** Swimming in 35⸰C water.

**HOMA-IR.** ANOVA revealed that the effect of groups was not significant (F _(6,48)_ = 0.124, p = 0.993, η^2^ = 0.017). However, a higher level of HOMA-IR is seen in DC group than HC (p > 0.05), and a lower level is seen in all experimental groups in comparison to DC (p > 0.05) (Fig. 3).

**Fig. 4:**
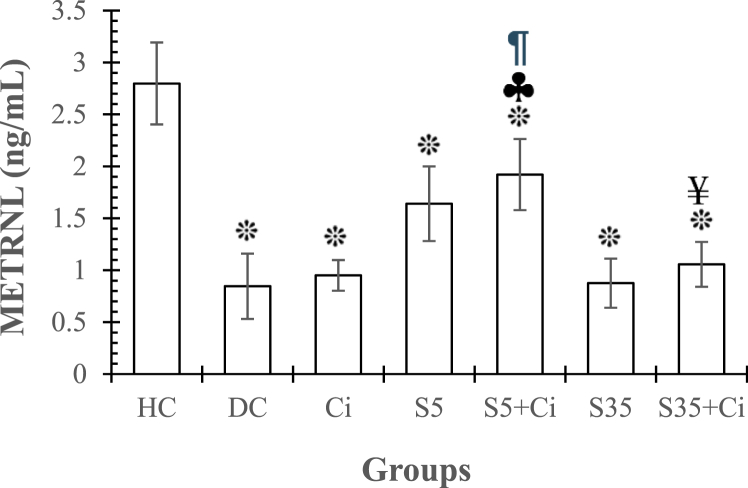
represents the Mean ± SE of METRNL in different groups. *: significant differences compared to the HC group. **♣:** significant differences compared to the DC group. **¶:** significant differences compared to the Ci group. **¥:** significant differences compared to the S5+Ci group. **HC:** Healthy Control. **DC:** Diabetic Control. **Ci:** Cinnamon feeding. **S5:** Swimming in 5^⸰^C water. **S35:** Swimming in 35^⸰^C water.

**METRNL.** ANOVA revealed that the effect of groups was significant (F _(6,48)_ = 5.92, p < 0.001, η^2^ = 0.485). LSD's post-hoc test results showed that the METRNL level in all groups (DC, Ci, S5, S5+Ci, S35 and S35+Ci, respectively) was significantly lower than in the HC group (p < 0.001, p < 0.001, p = 0.009, p = 0.044, p < 0.001, and p < 0.001). Additionally, the S5+Ci group exhibited a significantly higher METRNL level compared to the DC, Ci, and S35+Ci groups (p = 0.15, p = 0.027, and p = 0.047, respectively). Cinnamon consumption alone (Ci) had no effect on METRNL diabetic rats (p > 0.05). Swimming in cold water (S5) resulted in a 50 % increase in METRNL, but it was not significant (p > 0.05). Additionally, exercise in warm water (S35) did not affect MTRNL diabetic rats (p > 0.05) (Fig. 4).

**Fig. 5:**
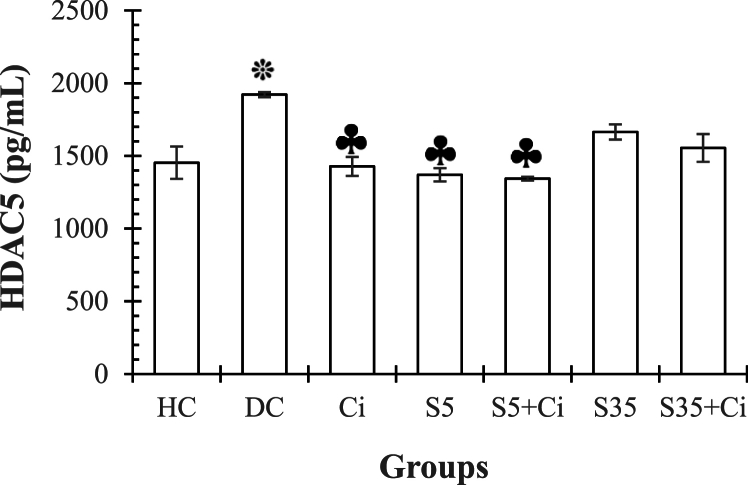
represents the Mean ± SE of HDAC5 in different groups. *: significant differences compared to the HC group. **♣:** significant differences compared to the DC group. **¶:** significant differences compared to the Ci group. **¥:** significant differences compared to the S5+Ci group. **HC:** Healthy Control. **DC:** Diabetic Control. **Ci:** Cinnamon feeding. **S5:** Swimming in 5^⸰^C water. **S35:** Swimming in 35^⸰^C water.

**HDAC5.** ANOVA revealed that the effect of groups was significant (F_(6,48)_ = 3.59, p = 0.045, η^2^ = 0.142). LSD's post-hoc test results showed that the HDAC5 level in the DC groups was higher than in the HC group (p = 0.048), and in the Ci, S5, and S35+Ci groups was significantly lower than in the DC group (p = 0.043, p = 0.038, and p = 0.029, respectively). No other comparisons demonstrated significant differences (p > 0.05) (Fig. 5).

## 4 Discussion

Exercise and herbal remedies are frequently utilized therapeutic modalities for managing diverse conditions linked to obesity and overweight, such as diabetes and other metabolic ailments. Nonetheless, our study findings revealed that the consumption of cinnamon and swimming in cold water as independent interventions did not lead to a noteworthy modification in METRNL levels. However, when combined, the consumption of cinnamon and swimming in cold water led to a notable increase in METRNL levels. The precise mechanisms underlying the changes in METRNL levels following cinnamon consumption remain unclear. However, based on the available evidence, the positive impact of cinnamon consumption may be attributed to enhancements in fat metabolism and an increase in γPPAR (gamma peroxisome proliferator-activated receptor) activity [32]. On the other hand, prior studies have suggested that exercise training leads to METRNL expression in skeletal muscles [8], while exposure to cold temperatures induces METRNL expression in white adipose tissue [33]. Concerning cold exposure and METRNL expression, studies have demonstrated that inhibiting METRNL actions in vivo attenuated cold-induced (4 °C) thermogenic responses. These findings suggest that METRNL is involved in the adaptive response to cold temperatures [33]. In line with our findings, Saghebjoo (2018) conducted a study on overweight individuals engaging in exercise under three temperature conditions (warm and cold temperate) and demonstrated that METRNL levels increased in response to exercise in water at moderate and warm temperatures. Conversely, the study revealed a decrease in METRNL levels following exercise in cold water [34]. It should be noted that the cold temperature range examined in Saghebjoo's study was approximately 16.5–17.5 °C. However, in our study, the ambient temperature was significantly lower, ranging from 4 to 5 °C. This substantial temperature difference could potentially account for the variations observed in the results [33].

Our study also revealed a significant decrease in HDAC5 levels in both the cold-water swimming group and the cold-water swimming + cinnamon consumption group. Additionally, all groups exhibited a significant reduction in fasting blood sugar (FBS) compared to the HC group. Previous research has demonstrated that aquatic exercise training leads to a notable decrease in FBS levels in individuals diagnosed with type 2 diabetes [[35], [36], [37]]. Besides, the ingredients in cinnamon have specific insulin properties [38]. One of the active ingredients derived from cinnamon is a polymer called methyl hydroxychalcone, which acts like insulin [39]. Cinnamon polyphenols stimulate glucose uptake like insulin and stimulate glycogen biosynthesis by activating the glycogen synthetase and inhibiting the action of glycogen synthetase kinase [40]. Peng et al. (2008) stated that the polyphenols of cinnamon prevent the formation of glycosylated end-products in the serum [41]. Mirfeizi et al. (2014) showed that FBG in T2DM patients receiving cinnamon decreased significantly instead of before the intervention [42]. In diabetic individuals, a 6-week endurance training program resulted in a significant reduction in HDAC5 levels [43]. Exercise has been demonstrated to activate AMPK through two pathways: the AMP-dependent pathway and the Ca^2+^-dependent pathway. These activations subsequently contribute to improved glucose homeostasis [44]. AMPK, through phosphorylation of HDAC5, enhances the transcription of GLUT4 [10,44]. Consequently, HDAC5 inhibitors have the potential to serve as a novel treatment for diabetes by increasing the expression of the GLUT4 gene [10].‬‬‬‬‬‬‬‬‬‬‬‬‬‬‬‬‬‬

In our study, both cinnamon consumption alone and cold-water swimming combined with cinnamon consumption did not yield a significant impact on insulin levels and insulin sensitivity index (HOMA-IR). In previous studies on cinnamon consumption alone or in combination with exercise, improvements in glycemic indices have been reported [23,24,32]. Research has demonstrated that cinnamon can effectively lower blood glucose levels and enhance insulin sensitivity in rats. These effects are attributed to increased insulin activity and improved glucose metabolism specifically within adipocytes [23,32]. Cinnamon inhibits the action of insulin receptor phosphatase and activates insulin receptor kinase in adipocytes. This mechanism leads to enhanced insulin sensitivity and reduced insulin resistance [23]. However, in the present study, no significant changes were observed in factors such as insulin and insulin resistance index in different groups. It has been suggested that the dosage of cinnamon employed might not be a contributing factor to the divergent outcomes observed across various studies. Moreover, the specific type of medication used by individuals with diabetes could potentially influence the outcomes of the study [24]. Furthermore, studies have reported that METRNL regulates insulin sensitivity through the gamma-activated receptor proliferator-activated receptor (γPPAR) pathway. METRNL acts as an insulin sensitizer and holds promising potential as a therapeutic target for addressing insulin resistance [45,46].

A review recently [47] stated that by raising intracellular calcium ion, reactive oxygen species (ROS), or AMP/ATP ratio levels in skeletal muscle cells, METRNL can activate signaling for AMP-activated protein kinase (AMPK) and peroxisome proliferator-activated receptor-δ (PPAR-γ). Activation of AMPK phosphorylation stimulated phosphorylation of HDAC5 and TBC1D1, which resulted in the activation and translocation of GLUT4 transcription from the cytoplasm to the membrane. PPAR-γ and AMPK phosphorylation that was highly expressed led to an elevation in fatty acid oxidation, IκBα phosphorylation, and NFκB nuclear translocation. Furthermore, METRNL promoted mitochondrial thermogenesis by increasing the expression of intramuscular PGC-1α and UCPs [47].

This study is the first evidence that stated the signaling of METRNL-HDAC5-Glucose uptake via cold exposure training (swimming) and cinnamon consumption.

The limitations of this study were a) the no assessment of GLUTE4 because of insufficient financial support, b) no report of HbA1C because of reporting in another study, and c) the insignificant decrease of HOMA-IR in experimental groups because of an insignificant reduction of insulin in the diabetic control group.

In summary, the findings from our study indicate a notable elevation in serum METRNL concentration in the cold-water swimming + cinnamon consumption group. Additionally, significant reductions in HDAC5 were observed in the cinnamon consumption group, cold-water swimming group, and cold-water swimming + cinnamon consumption group. Furthermore, all experimental groups displayed a significant decrease in fasting blood sugar (FBS) levels compared to the DC group. However, no significant changes were observed in insulin levels and the HOMA-IR index among the different groups, but experiments could help to control HOMA-IR to some extend in the diabetic model and it is important clinically. Based on these results, it is recommended that individuals perform cold-water swimming exercises with cinnamon consumption to improve diabetes-related indices.

## Funding statement

This work was taken from the research project and supported by the Allameh Tabataba'i University, Tehran, IRAN. [grant numbers: #T/D/10/51100,1394].

## Data availability

All data generated or analyzed during this study are included in this published article.

## Ethics approval statement

All the experimental procedures were performed according to the international care and use of laboratory animals' guidelines; All protocols of this research were approved by the Institutional Review Board of Allameh Tabataba'i University (#T/D/10/51100,1394).

## CRediT authorship contribution statement

**Seyed Morteza Tayebi:** Writing – original draft, Supervision. **Saleh Motaghinasab:** Writing – original draft, Methodology. **Rasoul Eslami:** Software, Formal analysis. **Somayeh Ahmadabadi:** Writing – review & editing, Data curation. **Aref Basereh:** Methodology. **Iman Jamhiri:** Methodology.

## Declaration of competing interest

The authors declare that they have no known competing financial interests or personal relationships that could have appeared to influence the work reported in this paper.

## References

[1] Saeidi A., Hackney A.C., Tayebi S.M., Ahmadian M., Zouhal H. (2019). Diabetes, insulin resistance, Fetuin-B and exercise training. Annals of Applied Sport Science.

[2] Tayebi S.M., Ghanbari-Niaki A., Saeidi A., Hackney A.C. (2017). Exercise training, neuregulin 4 and obesity. Ann Appl Sport Sci.

[3] Saeidi A., Tayebi S.M., Khosravi A., Razi O., Sellami M., Abderrahman A.B. (2019). Obesity, fat mass, osteopontin and exercise training. Int. J. Appl. Exerc. Physiol..

[4] Rao Kondapally Seshasai S., Kaptoge S., Thompson A., Di Angelantonio E., Gao P., Sarwar N. (2011). Diabetes mellitus, fasting glucose, and risk of cause-specific death. N. Engl. J. Med..

[5] Petersmann A., Müller-Wieland D., Müller U.A., Landgraf R., Nauck M., Freckmann G. (2019). Definition, classification and diagnosis of diabetes mellitus. Exp. Clin. Endocrinol. Diabetes.

[6] Kolahdouzi S., Baghadam M., Kani-Golzar F.A., Saeidi A., Jabbour G., Ayadi A. (2019). Progressive circuit resistance training improves inflammatory biomarkers and insulin resistance in obese men. Physiol. Behav..

[7] Dezhkam N., Rezaeian N. (2021). Effect of six weeks of aerobic training on meteorin like factor response and insulin resistance index in overweight and obese young women. Journal of Applied Health Studies in Sport Physiology.

[8] Tayebi S.M., Golmohammadi M., Eslami R., Shakiba N., Costa P.B. (2023). The effects of eight weeks of circuit resistance training on serum METRNL levels and insulin resistance in individuals with type 2 diabetes. J. Diabetes Metab. Disord..

[9] Saeidi A., Tayebi S.M., Khosravi A., Malekian F., Khodamoradi A., Sellami M. (2019). Effects of exercise training on type 2-diabetes: the role of Meteorin-like protein. Health Promot. Perspect..

[10] Lee J.O., Byun W.S., Kang M.J., Han J.A., Moon J., Shin M.J. (2020). The myokine meteorin‐like (metrnl) improves glucose tolerance in both skeletal muscle cells and mice by targeting AMPKα2. FEBS J..

[11] McGee S.L., Swinton C., Morrison S., Gaur V., Campbell D.E., Jorgensen S.B. (2014). Compensatory regulation of HDAC5 in muscle maintains metabolic adaptive responses and metabolism in response to energetic stress. Faseb. J..

[12] Tayebi S.M., Eslami R., Iranshad I., Golmohammadi M. (2023). The effect of eight weeks of circuit resistance training on serum levels of GPR119 and β-arrestin1 in individuals with type 2 diabetes. Ann Appl Sport Sci.

[13] Tayebi S.M., Saeidi A., Shahghasi R., Golmohammadi M. (2023). The eight-week circuit resistance training decreased the serum levels of WISP-1 and WISP-2 in individuals with type 2 diabetes. Ann Appl Sport Sci.

[14] Tayebi S.M., Hasannezhad P., Saeidi A., Fadaei M.R. (2018). Intense circuit resistance training along with zataria multiflora supplementation reduced plasma retinol binding protein-4 and tumor necrosis factor-α in postmenopausal females. Jundishapur J. Nat. Pharm. Prod..

[15] Tayebi S.M., Saeidi A., Fashi M., Pouya S., Khosravi A., Shirvani H. (2019). Plasma retinol-binding protein-4 and tumor necrosis factor-α are reduced in postmenopausal women after combination of different intensities of circuit resistance training and Zataria supplementation. Sport Sci. Health.

[16] Bae J.Y. (2018). Aerobic exercise increases meteorin-like protein in muscle and adipose tissue of chronic high-fat diet-induced obese mice. BioMed Res. Int..

[17] Garcia-Beltran C., Navarro-Gascon A., López-Bermejo A., Quesada-López T., de Zegher F., Ibáñez L. (2023). Meteorin-like levels are associated with active brown adipose tissue in early infancy. Front. Endocrinol..

[18] McGee S.L., Van Denderen B.J., Howlett K.F., Mollica J., Schertzer J.D., Kemp B.E. (2008). AMP-activated protein kinase regulates GLUT4 transcription by phosphorylating histone deacetylase 5. Diabetes.

[19] Czubryt M.P., McAnally J., Fishman G.I., Olson E.N. (2003). Regulation of peroxisome proliferator-activated receptor γ coactivator 1α (PGC-1α) and mitochondrial function by MEF2 and HDAC5. Proc. Natl. Acad. Sci. USA.

[20] Bukowiecki L.J. (1989). Energy balance and diabetes. The effects of cold exposure, exercise training, and diet composition on glucose tolerance and glucose metabolism in rat peripheral tissues. Can. J. Physiol. Pharmacol..

[21] Hanssen M.J., Hoeks J., Brans B., Van Der Lans A.A., Schaart G., Van Den Driessche J.J. (2015). Short-term cold acclimation improves insulin sensitivity in patients with type 2 diabetes mellitus. Nat. Med..

[22] Laursen T.L., Zak R.B., Shute R.J., Heesch M.W., Dinan N.E., Bubak M.P. (2017). Leptin, adiponectin, and ghrelin responses to endurance exercise in different ambient conditions. Temperature.

[23] Qin B., Panickar K.S., Anderson R.A. (2010). Cinnamon: potential role in the prevention of insulin resistance, metabolic syndrome, and type 2 diabetes. J. Diabetes Sci. Technol..

[24] Li X., Lu H.-Y., Jiang X.-W., Yang Y., Xing B., Yao D. (2021). Cinnamomum cassia extract promotes thermogenesis during exposure to cold via activation of brown adipose tissue. J. Ethnopharmacol..

[25] Hasanzade F., Toliat M., Emami S.A., Emamimoghaadam Z. (2013). The effect of cinnamon on glucose of type II diabetes patients. Journal of traditional and complementary medicine.

[26] Sengsuk C., Sanguanwong S., Tangvarasittichai O., Tangvarasittichai S. (2016). Effect of cinnamon supplementation on glucose, lipids levels, glomerular filtration rate, and blood pressure of subjects with type 2 diabetes mellitus. Diabetology international.

[27] Lubkowska A., Bryczkowska I., Gutowska I., Rotter I., Marczuk N., Baranowska-Bosiacka I. (2019). The effects of swimming training in cold water on antioxidant enzyme activity and lipid peroxidation in erythrocytes of male and female aged rats. Int. J. Environ. Res. Publ. Health.

[28] Bryczkowska I., Baranowska-Bosiacka I., Lubkowska A. (2017). Effect of repeated cold water swimming exercise on adaptive changes in body weight in older rats. Central European Journal of Sport Sciences and Medicine.

[29] Schreuder T.H.A., Green D.J., Nyakayiru J., Hopman M.T.E., Thijssen D.H.J. (2015). Time-course of vascular adaptations during 8 weeks of exercise training in subjects with type 2 diabetes and middle-aged controls. Eur. J. Appl. Physiol..

[30] Tinken T.M., Thijssen D.H., Black M.A., Cable N.T., Green D.J. (2008). Time course of change in vasodilator function and capacity in response to exercise training in humans. J. Physiol..

[31] Mustapha B.O., Ademoyegun O.T., Ahmed R.S. (2023). Horticultural crops as natural therapeutic plants for the therapy of diabetes mellitus. Egyptian Journal of Basic and Applied Sciences.

[32] Khan A., Safdar M., Ali Khan M.M., Khattak K.N., Anderson R.A. (2003). Cinnamon improves glucose and lipids of people with type 2 diabetes. Diabetes Care.

[33] Rao R.R., Long J.Z., White J.P., Svensson K.J., Lou J., Lokurkar I. (2014). Meteorin-like is a hormone that regulates immune-adipose interactions to increase beige fat thermogenesis. Cell.

[34] Saghebjoo M., Einaloo A., Mogharnasi M., Ahmadabadi F. (2018). The response of meteorin-like hormone and interleukin-4 in overweight women during exercise in temperate, warm and cold water. Horm. Mol. Biol. Clin. Invest..

[35] McGee S.L., Hargreaves M. (2010). Histone modifications and skeletal muscle metabolic gene expression. Clin. Exp. Pharmacol. Physiol..

[36] Espeland D., de Weerd L., Mercer J.B. (2022). Health effects of voluntary exposure to cold water–a continuing subject of debate. Int. J. Circumpolar Health.

[37] Cox K.L., Burke V., Beilin L.J., Puddey I.B. (2010). A comparison of the effects of swimming and walking on body weight, fat distribution, lipids, glucose, and insulin in older women—the Sedentary Women Exercise Adherence Trial 2. Metabolism.

[38] Solomon T.P.J., Blannin A.K. (2009). Changes in glucose tolerance and insulin sensitivity following 2 weeks of daily cinnamon ingestion in healthy humans. Eur. J. Appl. Physiol..

[39] Vanschoonbeek K., Thomassen B.J., Senden J.M., Wodzig W.K., van Loon L.J. (2006). Cinnamon supplementation does not improve glycemic control in postmenopausal type 2 diabetes patients. J. Nutr..

[40] Jarvill-Taylor K.J., Anderson R.A., Graves D.J. (2001). A hydroxychalcone derived from cinnamon functions as a mimetic for insulin in 3T3-L1 adipocytes. J. Am. Coll. Nutr..

[41] Peng X., Cheng K.W., Ma J., Chen B., Ho C.T., Lo C. (2008). Cinnamon bark proanthocyanidins as reactive carbonyl scavengers to prevent the formation of advanced glycation endproducts. J. Agric. Food Chem..

[42] Mirfeizi M., Mehdizadeh Tourzani Z., Mirfeizi S.Z., Asghari Jafarabadi M., Rezvani H., Shoghi M. (2014). Effects of cinnamon on controlling blood glucose and lipids in patients with type II diabetes mellitus: a double blind, randomized clinical trial. medical journal of mashhad university of medical sciences.

[43] Bagheri Ms, Valipour dehnou V., Hematfar A. (2019). Effects of six weeks endurance training on protein levels of GLUT4 and HDAC5 in soleus muscle in diabetic rats. Iran. J. Diabetes & Lipid Disord..

[44] Cherian P., Al Khairi I., Abubaker J., Abu-Farha M., Mohamed G., Yaseen H.M.A. (2019). Irisin, Meteorin-like Protein and Bone Remodelling Markers in Obesity and T2D.

[45] Li Z.-Y., Song J., Zheng S.-L., Fan M.-B., Guan Y.-F., Qu Y. (2015). Adipocyte Metrnl antagonizes insulin resistance through PPARγ signaling. Diabetes.

[46] Jung T.W., Lee S.H., Kim H.-C., Bang J.S., Abd El-Aty A., Hacımüftüoğlu A. (2018). METRNL attenuates lipid-induced inflammation and insulin resistance via AMPK or PPARδ-dependent pathways in skeletal muscle of mice. Exp. Mol. Med..

[47] Li Z., Gao Z., Sun T., Zhang S., Yang S., Zheng M. (2023). Meteorin-like/Metrnl, a novel secreted protein implicated in inflammation, immunology, and metabolism: a comprehensive review of preclinical and clinical studies. Front. Immunol..

